# GM1a ganglioside-binding domain peptide inhibits host adhesion and inflammatory response of enterotoxigenic *Escherichia coli* heat-labile enterotoxin-B in HCT-8 cells

**DOI:** 10.1038/s41598-023-44220-5

**Published:** 2023-10-06

**Authors:** Jun-Young Park, Fukushi Abekura, Seung-Hak Cho

**Affiliations:** 1 Division of Zoonotic and Vector Borne Disease Research, Center for Infectious Disease Research, Korea National Institute of Health, Cheongju, Republic of Korea; 2 Environmental Diseases Research Center, Korea Research Institute of Bioscience and Biotechnology, Daejeon, Republic of Korea

**Keywords:** Bacteria, Pathogens, Microbiology, Diseases

## Abstract

Enterotoxigenic *Escherichia coli* (ETEC) is a major cause of illness and death but has no effective therapy. The heat-labile enterotoxin LT is a significant virulence factor produced by ETEC. The heat-labile enterotoxin-B (LT-B) subunit may enter host cells by binding to monosialotetrahexosylganglioside-a (GM1a), a monosialoganglioside found on the plasma membrane surface of animal epithelial cells. This research was conducted to develop conformationally comparable peptides to the carbohydrate epitope of GM1a for the treatment of ETEC. We used the LT-B subunit to select LT-B-binding peptides that structurally resemble GM1a. The ganglioside microarray and docking simulations were used to identify three GM1a ganglioside-binding domain (GBD) peptides based on LT-B recognition. Peptides had an inhibiting effect on the binding of LT-B to GM1a. The binding capacity, functional inhibitory activity, and in vitro effects of the GBD peptides were evaluated using HCT-8 cells, a human intestinal epithelial cell line, to evaluate the feasibility of deploying GBD peptides to combat bacterial infections. KILSYTESMAGKREMVIIT was the most efficient peptide in inhibiting cellular absorption of LT-B in cells. Our findings offer compelling evidence that GM1a GBD-like peptides might act as new therapeutics to inhibit LT-B binding to epithelial cells and avoid the subsequent physiological consequences of LT.

## Introduction

Enterotoxigenic *Escherichia coli* (ETEC) is responsible for the production of heat-labile enterotoxin (LT). ETEC causes for more than 50,000 fatalities and millions of cases of diarrhea each year^1^. Despite advances in sanitary facilities, the death and morbidity rates of ETEC are not decreasing. When children are infected with ETEC, it often results in long-term health issues such as stunted development and decreased cognitive ability, which may start a vicious cycle of poverty^2^. Additionally, travelers visiting endemic locations, especially medical and military professionals, are susceptible to contracting ETEC. ETEC has also been associated with chronic disorders such as irritable bowel syndrome^3^. Watery diarrhea caused by the enterotoxin helps the illness spread via the fecal–oral pathway, which is exacerbated by the infection.

LT belong to the AB5 toxin family, which is made up of five B subunits and one A subunit overall^4,5^. The B-torus-shaped pentamer’s core, which is responsible for binding to epithelial cells, is connected to the catalytically active A subunit^6^. These two components work together to form the protein complex. With more than 80% sequence similarity, the AB5 toxins cholera toxin (CT) and *E. coli* fever susceptible enterotoxin are physically and functionally identical^7^. Despite having a similar structure, the B pentamer differs in the precise binding location for gangliosides in the cell membrane by roughly 20%. Various mammalian species have diverse species specific cell surface glycan in intestinal epithelial cells^8^. Among them, Gb3 and GM1 are well known glycosphingolipids, which exist in human intestinal environment^9,10^. Gb3 is known as Shiga toxin receptor in human intestinal epithelial cell^11^. Some studies reported that N-terminal alpha helix sustains conformation of the GM1 binding pocket as essential factor. Several studies reported that GM1 mimic peptide inhibited both of cholera toxin binding and uptake and CT binding inhibition has been reported in the event that sialic acid in GM1 is oxidized^12–14^. The B pentamer (CTB) of CT has been demonstrated in several investigations to bind to the monosialotetrahexosylganglioside GM1 ganglioside; however, heat-labile enterotoxin-B (LT-B) does not share the same binding site as CT^15^. Studies on the behavior of peptides that bind to and inhibit certain sequences of GM1a in the instance of LT-B are less well known. Compared to CT, LT is often more promiscuous and can bind to a larger range of glycosphingolipids^16,17^. In many significant molecular interactions between proteins and glycolipids, one or more monosaccharides are glycosidically attached to a lipid moiety in a molecule known as a glycolipid^18,19^. The oligosaccharide moiety is often accessible but near the bilayer surface because the lipid moiety is typically buried in the cell membrane lipid bilayer^20,21^, which offers a special setting for interactions between proteins and carbohydrates^22^. This includes intestinal polyglycosylceramides, sialic-acid-containing glycosphingolipids, disialoganglioside GD2, GM1, and lacto-N-neotetraosylceramide (LNnT-Cer; paragloboside)^23,24^. Relevant previous studies include the results of LT-B's GM1a binding site, Gly33^25^, and the structural basis for differential receptor binding of cholera and *E. coli* heat-labile toxins^26^.

Herein, we demonstrated that GM1a-like peptides are capable of imitating GM1a by blocking LT-B binding to GM1a. We were able to get three peptides that were successful in inhibiting the binding of LT-B to GM1a by using the ganglioside microarray^27^ and docking simulations^28^. The peptides offer enormous potentials for the treatment of cholera and other bacterial illnesses that employ GM1a as a receptor, and they show promise as a valuable tool for competing with LT-B for GM1a. The clinical application of the findings from this line of study ought to be of assistance to individuals who are afflicted with this and other ailments caused by comparable microorganisms.

## Materials and methods

### Glycan microarray screening

The robotic non-contact spotter, sciFLEXARRAYER S11 from Scienion AG (Berlin, Germany), was utilized to print ganglioside microarrays on Nexterion® H NHS activated glass slides (Schott AG, Mainz, Germany). The microarray comprised 20 different glycans and was used to assess whether the LT toxin lectin domains exhibit variations in their recognition of glycan receptors, thereby determining tropism. To detect the binding of LT toxin (200 µg/mL), anti-LT toxin polyclonal rabbit IgG (10 µg/mL) was used, followed by fluorescently labeled goat anti-rabbit polyclonal antibodies (IgG-488, 5 µg/mL). The spot pitches of 550 µm were employed to spatially align droplets (2.5 nL) of 50 nM ganglioside GM1a in sodium phosphate buffer (300 mM, pH 8.5, 0.005% Tween 20). After printing, the slides were exposed for 18 h to a saturated NaCl solution at 75% humidity and 25 °C. Subsequently, the slides were cleaned in PBST (0.5% Tween 20 in PBS solution), PBS, and water before being dried on a slide spinner. To quantify fluorescence, a GenePix 4300/4400 Microarray Scanner (Molecular Devices, CA, USA) was employed.

### Docking simulation

Docking simulation enabled us to decipher the structure of lectin in the pathogen and identify the site of attachment to the host’s receptor. Using SWISS-MODEL, a homology modeling approach, the three-dimensional (3D) structure of LT (PDB ID: 1LTS) was produced using a GM1a (PDB ID: 6LF2) 3D structure. The docking simulation was conducted using the 3D structure of GM1a. The Chem-office application (http://www.cambridgesoft.com, version: 7.0) was used to reduce energy. Autodock Vina 1.1.2 was used for the docking simulations. Possible hydrogen bonds and hydrophobic interactions were identified utilizing HBPLUS and non-bonded contact parameters as default settings in a LigPlot based on the findings of docking simulation.

### Solid phase peptide synthesis and FITC labeling

On Rink Amide MBHA resin (loading capacity: 0.54 mmol/g), peptide synthesis was manually carried out using a conventional Fmoc-based SPPS. Rink amide AM resin was typically pre-swollen with DMF for an hour. With 50% morpholine (in DMF), Fmoc was deprotected for 30 min. The resin was then washed five times each with DMF, DCM, and DMF (5 times). Following the first resin loading, HCTU (4.9 equiv) and Fmocprotected amino acids (5.0 equiv) were dissolved in NMP before DIPEA (10.0 equiv). After the combination was introduced to the resin for two hours and pre-activated for one minute, the resin was washed five times with DMF, five times with DCM, and five times with DMF (5 times). For FITC labeling, the resin was exposed to a solution of FITC (5.0 equiv based on the resin's initial loading) and DIPEA (10.0 equiv) dissolved in DMF overnight in the dark. Then, peptides were separated from the resin using a TFA/H2O/EDT/TIS (94:2.5:2.5:1) combination for two hours before being concentrated. The FITC-labeled peptides were then dissolved in water and acetonitrile, and their amounts were determined by measuring their absorbance at 494 nm after being precipitated with hexane and et2o (1:1 in volume) at -20 °C. As in the above procedure, peptide synthesis proceeded with the same protocol for P1 (KILSYTESMAGKREMVIIT), P2 (SYTESMAGKRE), and P3 (TLRITYLTETK).

### Peptide binding and inhibition assays

On a 96-well flat-bottom polystyrene microtiter plate, we assessed the inhibitory avidity and specificity of GM1a-GBD peptides for GM1a binding to LT-B. The ELISA plate's wells were coated with 25 ng of GM1a dissolved in 100% ethanol. Nonspecific binding sites in the wells were blocked using 100 mL of washing buffer (1% BSA in PBS) for 30 min at RT after the solvent was evaporated in an incubator at 37 °C. Using the EL 50 microplate strip washer, the plate was washed five times with 300 mL of the washing buffer after being decanted of the blocking solution (Thermo fisher scientific Massachusetts, U.S.). A reaction solution of LT-B, HRP-conjugated LT-B (HRP-CTB) 1:20,000, including a GM1a- GBD peptide P1, P2 and P3(10, 100 and 1000 nM) was added to the plate after further washing for 2 h at room temperature. After washing the plate with the aforementioned washing buffer, a chromogenic reagent [100 mL of o-phenylenediamine dihydrochloride (OPD) peroxidase substrate in PBS; Sigma-Aldrich] was added. After 2 min of dark incubation, the reaction was stopped by adding 50 mL of 3 N sulfuric acid to the plate. Each well's absorbance was assessed using a SpectraMax iD5 Microplate Reader at 492 nm (Molecular Devices, San Jose, U.S.).

### Cell culture and MTT assay

The HCT-8 cell line, obtained from the American Type Culture Collection (ATCC, Rockville, MD, United States), was cultured in DMEM supplemented with 10% FBS and 1% antibiotic–antimycotic solution (Gibco, USA). The cells were grown in a humidified atmosphere with 5% CO_2_ at 37 °C. To assess cell viability, HCT-8 cells were seeded in a 96-well plate at a density of 1 × 10^4^ cells/well and exposed to different concentrations (0, 10, 100, and 1,000 nM) of P1. The MTT solution was then added and incubated at 37 °C in a CO_2_ incubator for 4 h. After solubilizing the reaction product with dimethyl sulfoxide, the optical density was measured at 550 nm following 15 min of incubation.

### Nitric oxide (NO) assay

HCT-8 cells were seeded in 24-well plates at a density of 1 × 10^5^ cells/well. They were then treated with LT-B alone or in combination with 1,000 nM of P1 for a duration of 24 h. After the incubation period in a CO_2_ incubator at 37 °C, the production of nitric oxide (NO) was evaluated by measuring the nitrite level in the culture media. This was achieved by mixing the medium with Griess reagent and measuring the optical density at 540 nm after 10 min of incubation.

### Measurement of pro-inflammatory cytokines and prostaglandin E2

Levels of pro-inflammatory cytokines, such as TNF-α, IL-6, and IL-1β, in culture media were assessed using ELISA kits (Affymetrix, eBioscience) with each cytokine-specific antibody following the manufacturer’s recommended protocol. The kit's microwells have been pre-coated with monoclonal antibodies that are each specific to IL-1beta, L-6, IL-8, and TNF-alpha. Following the addition of standards or samples, the strips will receive the biotin-conjugated detection antibody combination. The unbound components of the sample are fully removed from the microtiter plate wells. In each microplate well, Avidin conjugated to Horseradish Peroxidase (HRP) is added and incubated in order to quantify the quantity of cytokine present in the sample. To completely get rid of any unbound HRP-conjugated Avidin, the wells are carefully cleaned. Each well receives a TMB (3, 3', 5, 5' tetramethyl-benzidine) substrate solution. In a brief incubation time, the enzyme (HRP) and substrate are allowed to react. The quantity of a particular cytokine present in each well influences how strongly the color develops. By adding a sulphuric acid solution, the enzyme–substrate process is stopped, and the color turns yellow. A wavelength of 450 nm + /- 2 nm is used to spectrophotometrically quantify the intensity. Standards that had been diluted with a comparable matrix were examined alongside samples. The operator can then generate an optical density (O.D.) vs cytokine concentration (pg/mL) graph. The O.D. of the samples is then compared to the standards to determine the concentration of cytokines in the samples. Also, PGE_2_ production in the cell culture medium was evaluated with the same principle and method as above by an ELISA assay kit (Cayman Chemical) according to the manufacturer's recommended protocol.

## Statistical analysis

All experiments were carried out at least three times, and representative results are shown. The outcomes of data analyses were performed using GraphPad Prism software and were statistically analyzed using the comparison-based one-way analysis of variance and followed by post-hoc Bonferroni test to determine significance. Differences were considered statistically significant when p-values were < 0.05. *p indicates < 0.05 and **p < 0.01. The differences between the two figures are indicated in the figure legends.

## Results

### Finding lectin receptor of LT toxin linked to GM1a ganglioside using ganglioside microarray

**Figure 1:**
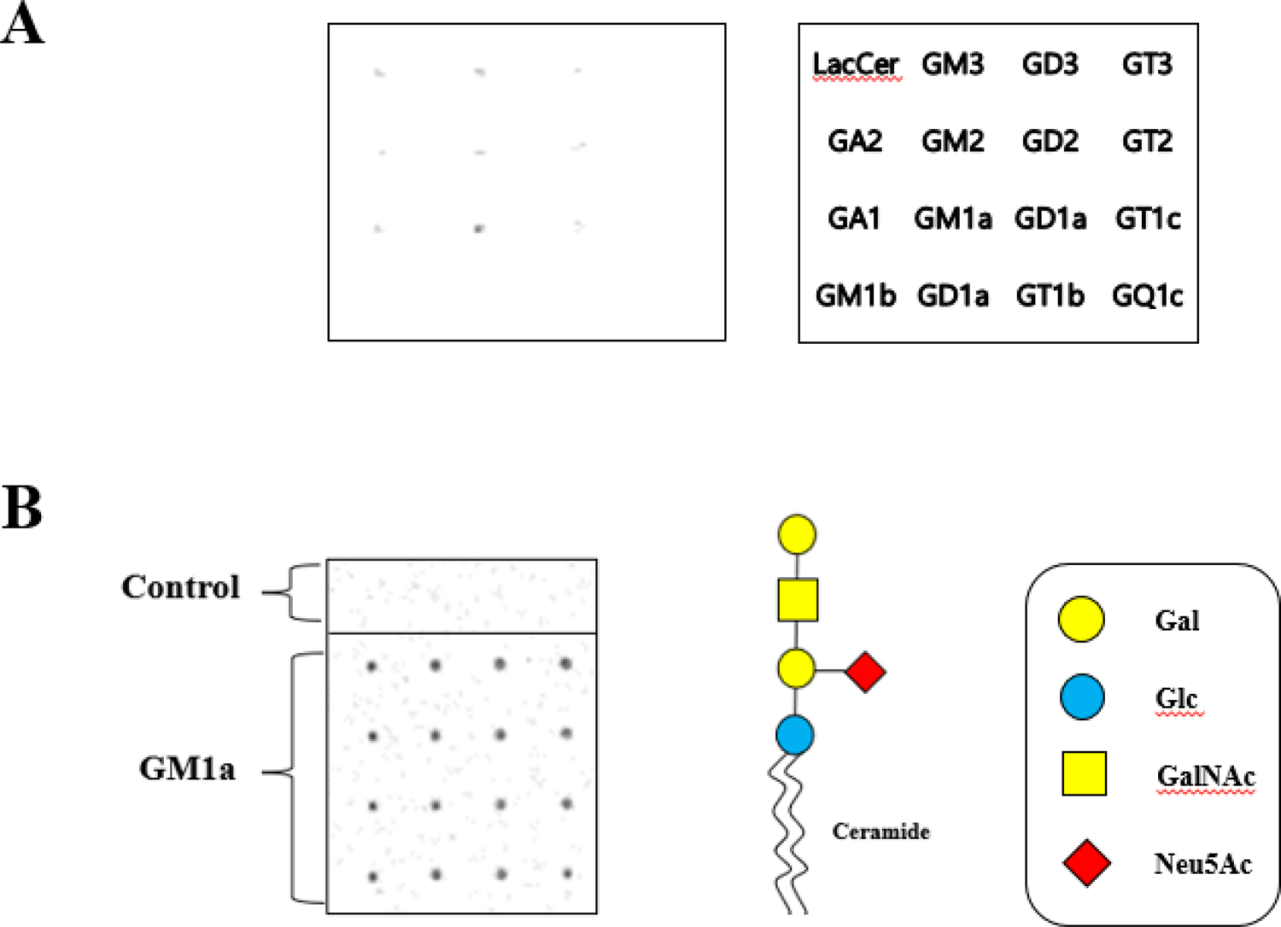
Finding lectin receptors of GM1a ganglioside-linked LT toxins using ganglioside microarray screening. (**A**) Screening ganglioside microarray experiments for specific binding of LT toxin lectin domains to 20 major gangliosides. The ganglioside microarray revealed lectin factors linked to GM1a ganglioside in LT toxin of enterotoxigenic *Escherichia coli*. (**B**) The lectin factors associated with GM1a ganglioside were identified using ganglioside microarray. The figure on the right shows the structure of GM1a ganglioside.

We conducted an investigation using 20 different glycans (in the mammalian printed array version) to explore domains in the recognition ranges of the LT toxin lectin. Our goal is to identify and screen several glycan receptors that specifically bind with LT toxins. In Fig. 1A, we screened for specific binding of the LT toxin lectin domain to 20 major gangliosides. After confirming that the LT toxin binds to GM1a, in the following experiments the printed sequences contained exclusively repeats of the glycan for LT and GM1a. The results clearly demonstrated the consistent and strong binding affinity of the end glycan (Fig. 1B). An experiment was conducted using a glycan microarray method to find the specificity of the toxin’s lectin-carbohydrate interaction, which revealed lectin factors linked to GM1a. The detailed process is described in the Materials and Methods section. Thick spots were selected and sequenced. As a result, LT-B toxin lectin was mainly detected, and a subsequent experiment was conducted using LT-B toxin lectin.

### Determination of GM1a ganglioside-binding domain (GBD) of LT-B by using docking simulations

**Figure 2:**
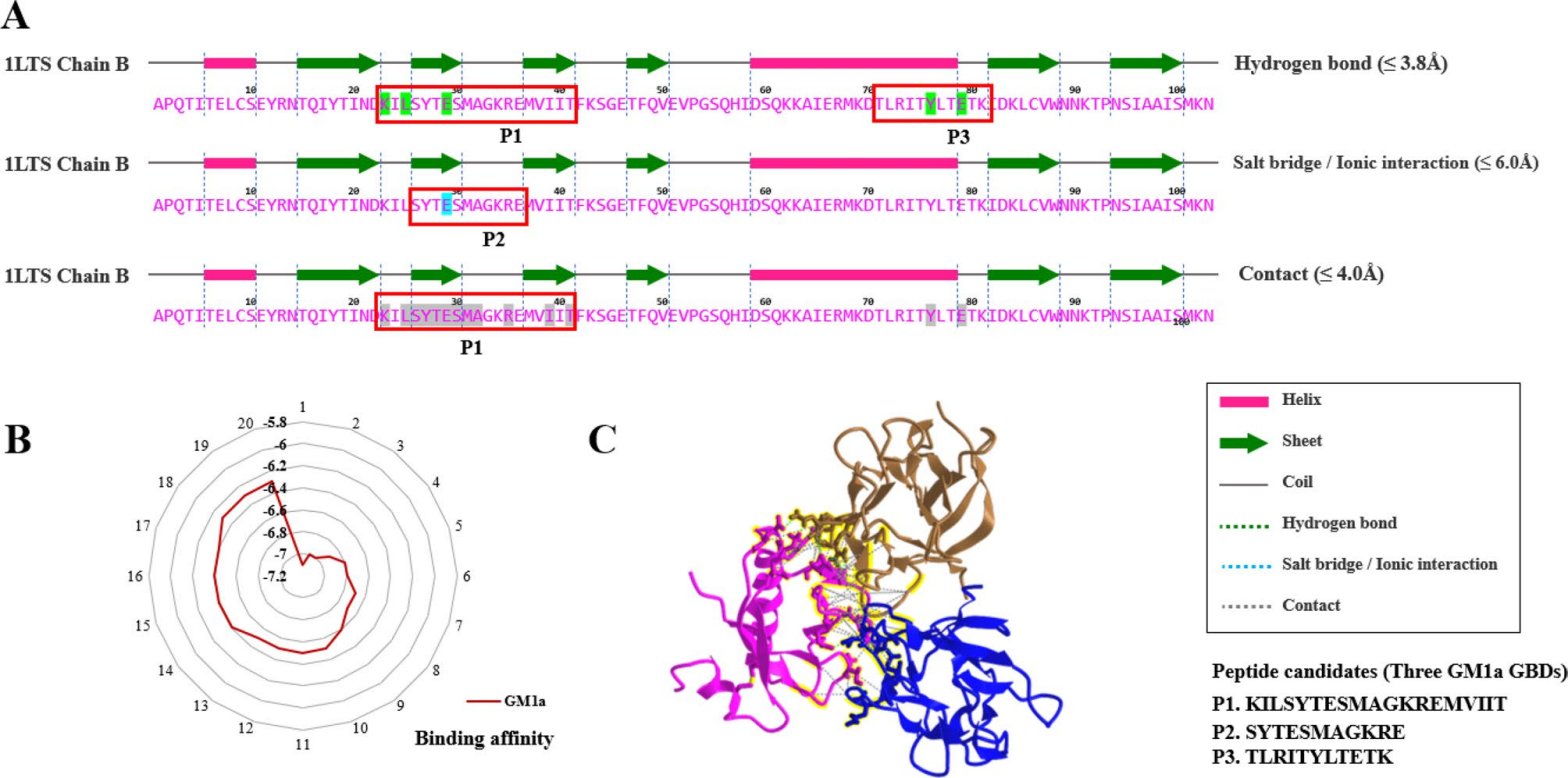
Docking simulations with LT-B, a lectin candidate that binds to GM1a glycan, were used to determine binding affinity. (**A**) Binding affinity was determined using docking simulations with LT-B, a lectin candidate that binds to GM1a glycan. Three GM1a ganglioside-binding domains were identified: hydrogen bonding, ionic bonding, and predicted binding sites (P1, P2 and P3). (**B**) Binding strength to GM1a glycan and LT-B. The unit of bond strength is kcal/mol. Numbers 1–20 mean the number of docking simulation bonding trials, and all 20 trials (*n* = 20) confirmed that the binding force with GM1a was ≤ -6 kcal/mol. (**C**) LT-B binds to GM1a and binding motif.

The GBD of LT-B that binds to GM1a ganglioside was determined by using docking simulations with LT-B, which is a lectin candidate that binds to GM1a ganglioside. To find the GM1a GBD of LT-B, we identified residues that had a binding affinity of ≤  − 6 kcal/mol for the LT-B residue interacting with the ligand, and ≤  − 6 kcal/mol or less for other ligands. This was performed for all ligands of LT-B and analyzed for hydrogen bonding, ionic bonding, and general bonding strength, respectively (supplemental data 1A-D). Subsequently, three GM1a GBDs (KILSYTESMAGKREMVIIT, SYTESMAGKRE, and TLRITYLTETK) were found at the expected binding sites (Fig. 2A,B). As for the predicted binding site, the amino acid sequence in the region where all three types of binding were high was selected as a candidate based on their respective binding strengths. The detailed combination numbers for each are shown in the supplementary material. The amino acid sites (KILSYTESMAGKREMVIIT, SYTESMAGKRE, and TLRITYLTETK) expected to bind through docking simulation are marked in yellow. The sequences of the three GBDs are all potential candidates for binding sites and have been named P1, P2, and P3, respectively.

### A comparison of the ability of GM1a GBD peptides to inhibit binding and compare their binding affinity

**Figure 3:**
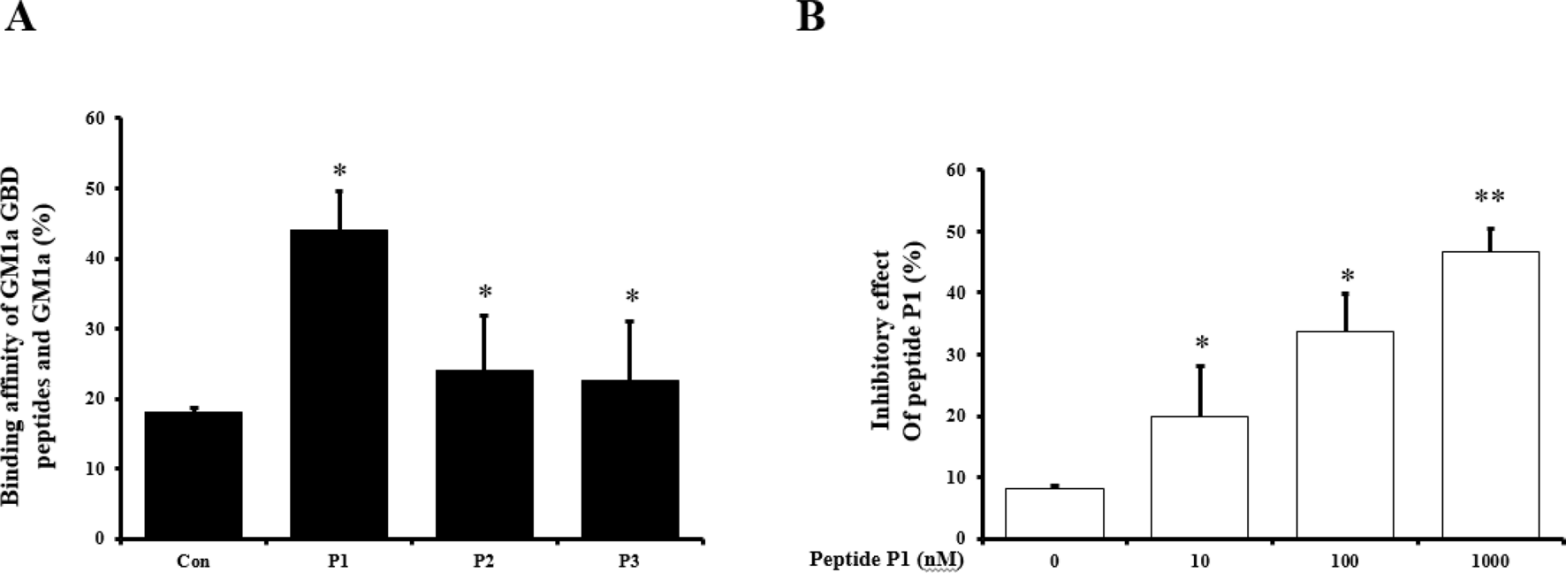
A comparison of the ability of GM1a GBD peptides to inhibit binding and compare their binding affinity. (**A**) Binding affinity to GM1a was compared among the GM1a GBD replica peptides. The term “control” refers to the binding affinity of a matrix without peptides. When compared to the Control, P1–P3 had statistically significant differences. (**B**) In vitro treatment with P1 inhibited activity in a concentration-dependent manner. GBD peptide P1 was treated with compounds at 0, 10, 100, and 1,000 nM. When compared to the Control, P1–P3 had statistically significant differences and represent three independent tests (*n* = 3). *Means were substantially different (*P < 0.05 and **P < 0.01).

Following the completion of our investigation, we found three GM1a GBDs. The peptide sequences P1, P2 and P3 are all potential candidates for the binding site. Examination of the adhesion using LT-B showed that P1, which is a GM1a GBD peptide, had the highest binding strength compared to the other peptides. P1, P2, and P3 all had significantly different levels of binding affinity to LT-B when compared with the control (Fig. 3A). We hypothesized that the GM1a GBD-like peptide would bind to LT-B and influence how it interacts with host glycans. Furthermore, as can be shown in Fig. 3B, in vitro treatment with peptide P1 decreased binding activity in a concentration-dependent manner. P1 was administered to LT-B at concentrations of 0, 10, 100, and 1,000 nM. According to the findings, LT-B inhibition was greater when a higher inhibitor concentration was administered.

### Effects of GM1a GBD peptide P1 on cell viability and production of PGE_2_ and nitric oxide (NO) in HCT-8 cells

**Figure 4:**
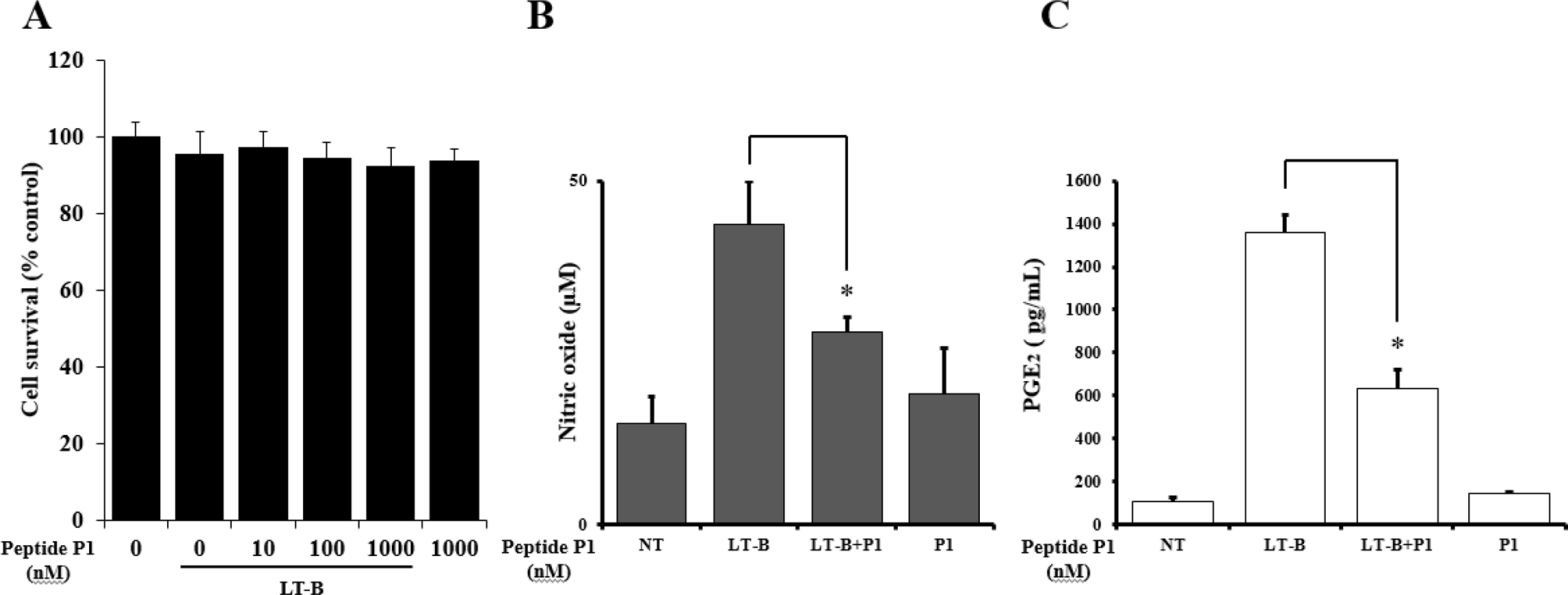
Cell viability on P1 and its inhibitory effect on PGE_2_ and nitric oxide (NO) production. (**A**) Cell viability measured by MTT assay. 1 × 10^4^ cell/well cells were treated with 0, 10, 100 and 1,000 nM of P1 and LT-B (100 nM) for 24 h. (**B**) 5 × 10^5^ cells/well cells were treated with LT-B (100 nM) alone or with 1,000 nM P1 for 24 h. NO in the medium was assessed using Griess assays. (**C**) 5 × 10^5^ cells/well cells were treated with LT-B (100 nM) alone or with 1,000 nM P1 for 24 h. PGE_2_ in the medium was assessed by ELISA. The results shown are mean ± SEM and represent three independent tests (*n* = 3). *P < 0.05 = significant differences from the LT-B treated cells.

The effects of GM1a GBD peptide P1 on the viability of HCT-8 cells in the presence or absence of LT-B was assessed. P1 did not cause significant cytotoxicity at concentrations of up to 1,000 nM with or without of LT-B (Fig. 4A). Moreover, its anti-inflammatory effects were tested at a concentration of 1,000 nM. Next, the effects of P1 were investigated by evaluating levels of NO and PGE_2_ production after LT-B activation as described in the Materials and Methods section. Treating HCT-8 cells with P1 without LT-B stimulation did not affect the production of NO or PGE_2_. However, P1 inhibited LT-B-stimulated production of PGE_2_ and NO (Fig. 4B,C, respectively). Thus, the inhibitory activities of P1 on NO and PGE_2_ production were examined at a concentration of 1,000 nM.

### Production of LT-B inflammatory cytokines by LT-B induced HCT-8 cells was suppressed by GM1a GBD peptide P1

**Figure 5:**
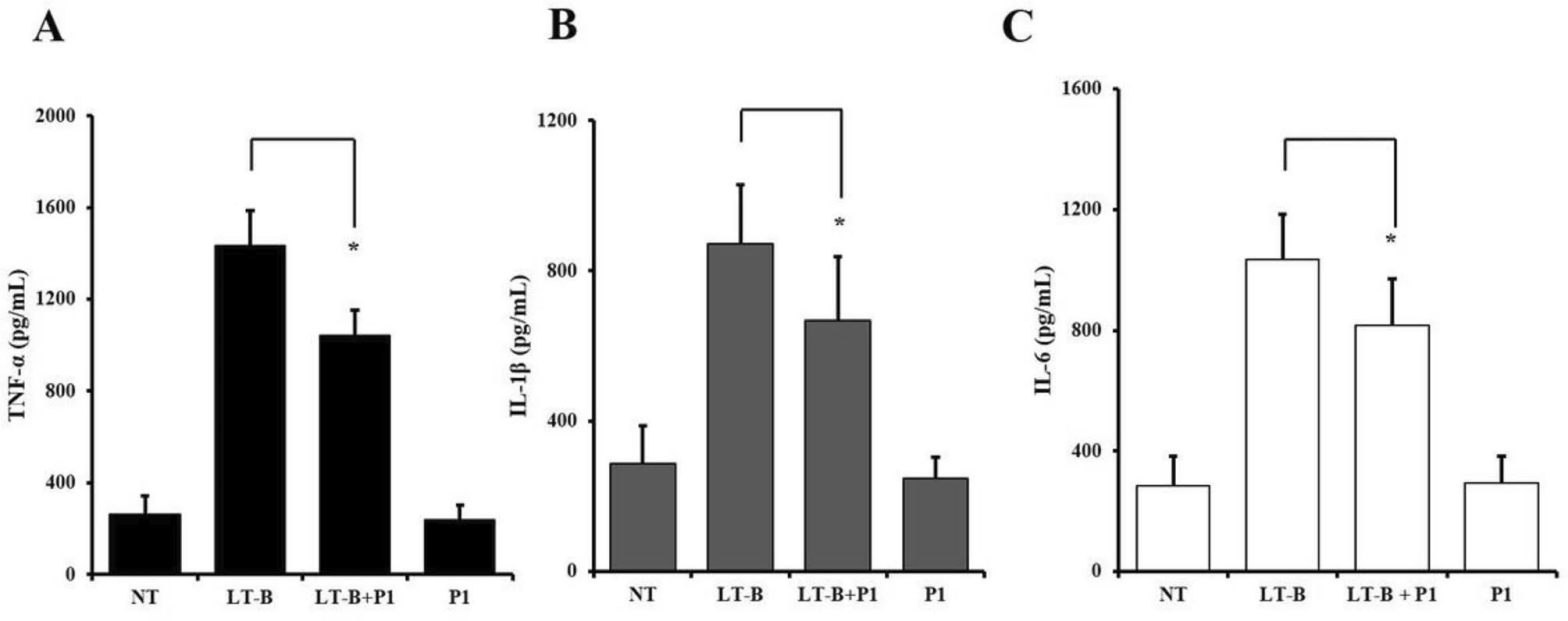
The production of cytokine by HCT-8 cells-induced LT-B was suppressed by peptide P1. (**A**), (**B**) and (**C**) 1,000 nM P1 treatment dose-dependently decreased LT-B-induced production of inflammatory cytokines TNF-α, IL-1β, and IL-6 by ELISA. The results shown are mean ± SEM and represent three independent tests (*n* = 3). *p < 0.05 = significant differences from the LT-B treated cells. NT, no treatment; LT-B, LT-B treatment; LT-B + P1, LT-B and P1 co-treatment; P1, P1 treatment.

Following a challenge infection with intestinal epithelial cell line HCT-8, cytokine levels were measured using ELISA to detect the impact of P1. After infection, levels of key inflammatory factors TNF-α, IL-6, and IL-1β were measured, and the results revealed that levels of all three inflammatory markers decreased (Fig. 5A−C). These findings provided conclusive evidence that P1 could inhibit host adhesion, interact with GM1a on the cell surface, and neutralize it when present in the culture medium.

### Inhibitory effect of GM1a GBD peptide P1 on spindle in LT-B-activated HCT-8 cells

The effects of GM1a-GBD peptide P1 on spindle in HCT-8 cells were examined. When HCT-8 cells are activated after challenge infection, the dendrites lengthen and change shape, resulting in stronger adhesion^29,30^. We investigated whether P1 inhibits these changes. While LT-B treatment stimulated dendrite growth, spindle was attenuated by P1 (Fig. 5). These data show that peptide P1 inhibits dendrite extension in LT-B-induced HCT-8 cells, and that peptide P1 inhibits dendrite elongation of HCT-8 cells after challenge infection. Additionally, peptide P1 and regulates activation before inflammatory reaction occurs.

## Discussion

Host cells, such as plants and animals, and microorganisms, such as bacteria, viruses, and fungi, contain carbohydrate chains and lectins that mutually identify one another^31–34^. When microbial pathogen-associated molecular patterns other than self-patterns, including gram-positive and -negative bacteria and fungi, are recognized in hosts, the defense mechanism is triggered^35,36^. Glycan-based PAMPs are associated with a group of lectins that are found in many eukaryotes^37,38^. The pathogen’s lectin-like proteins attach to the glycan-based outer membrane of host cells as the initial stage of bacterial infection in humans^39,40^. These interactions are typical for viruses, bacteria that adhere to surfaces through surface adhesion molecules, and bacteria that release toxins^41^. Bacterial toxins frequently have an enzyme that changes the physiology of the target cell by cytotoxicity and a binding domain that facilitates certain cellular interactions^42^. In recognition processes, such as immunological recognition events, bacterial and viral invasion and tissue development and repair cell surface glycans that are glycoconjugates play crucial roles as receptors^43–45^. However, to create effective therapeutic and diagnostic approaches, our knowledge of these biological processes at the molecular level must be greatly expanded.

**Figure 6:**
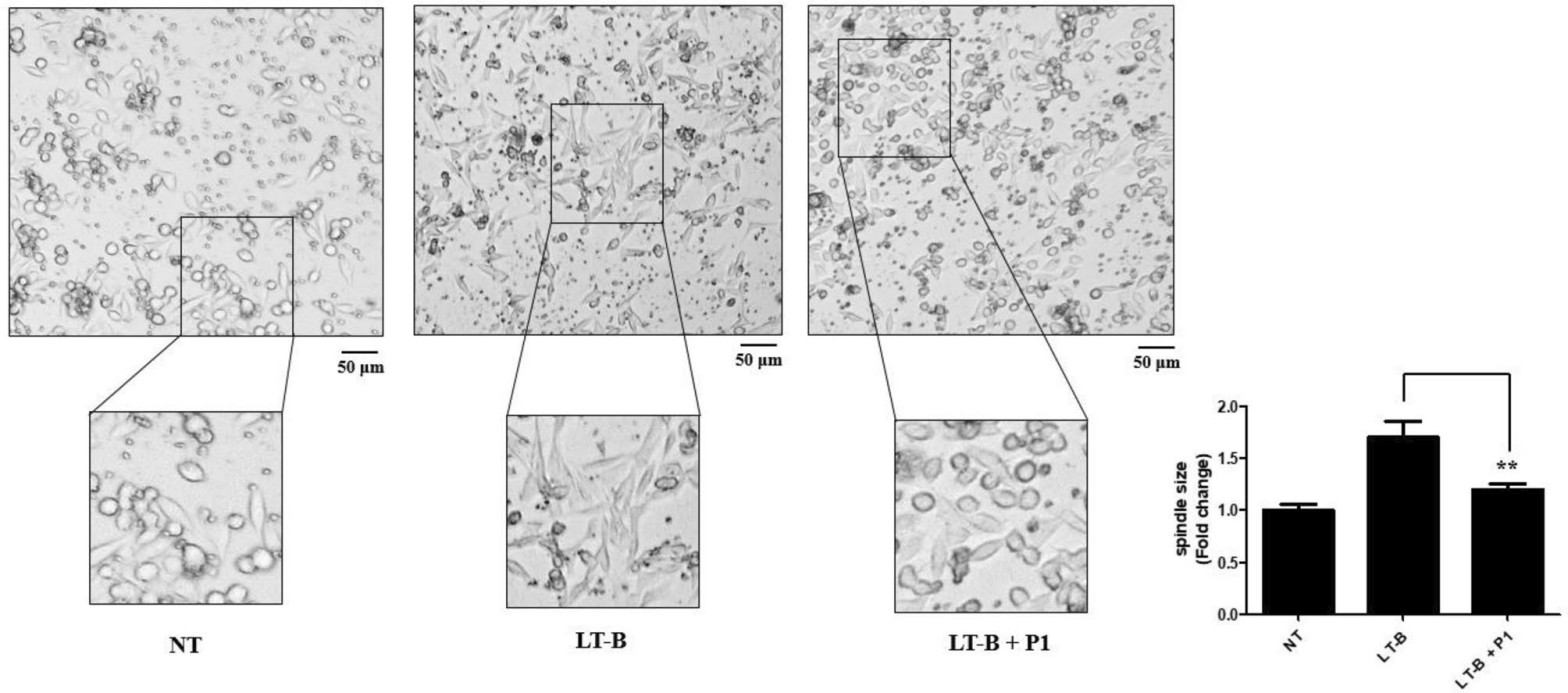
Inhibitory effect of P1 on spindle in LT-B-activated HCT-8 cells. Inhibitory effect of dendrite extension by P1 treatment in HCT-8 cells. Cells were treated with P1 at 1,000 nM. Cell morphology of micrograph (scale bar is 50 μm). NT, no treatment; LT-B, LT-B treatment; LT-B + P1, LT-B and P1 co-treatment. *Means were substantially different (**P < 0.01).

**Figure 7:**
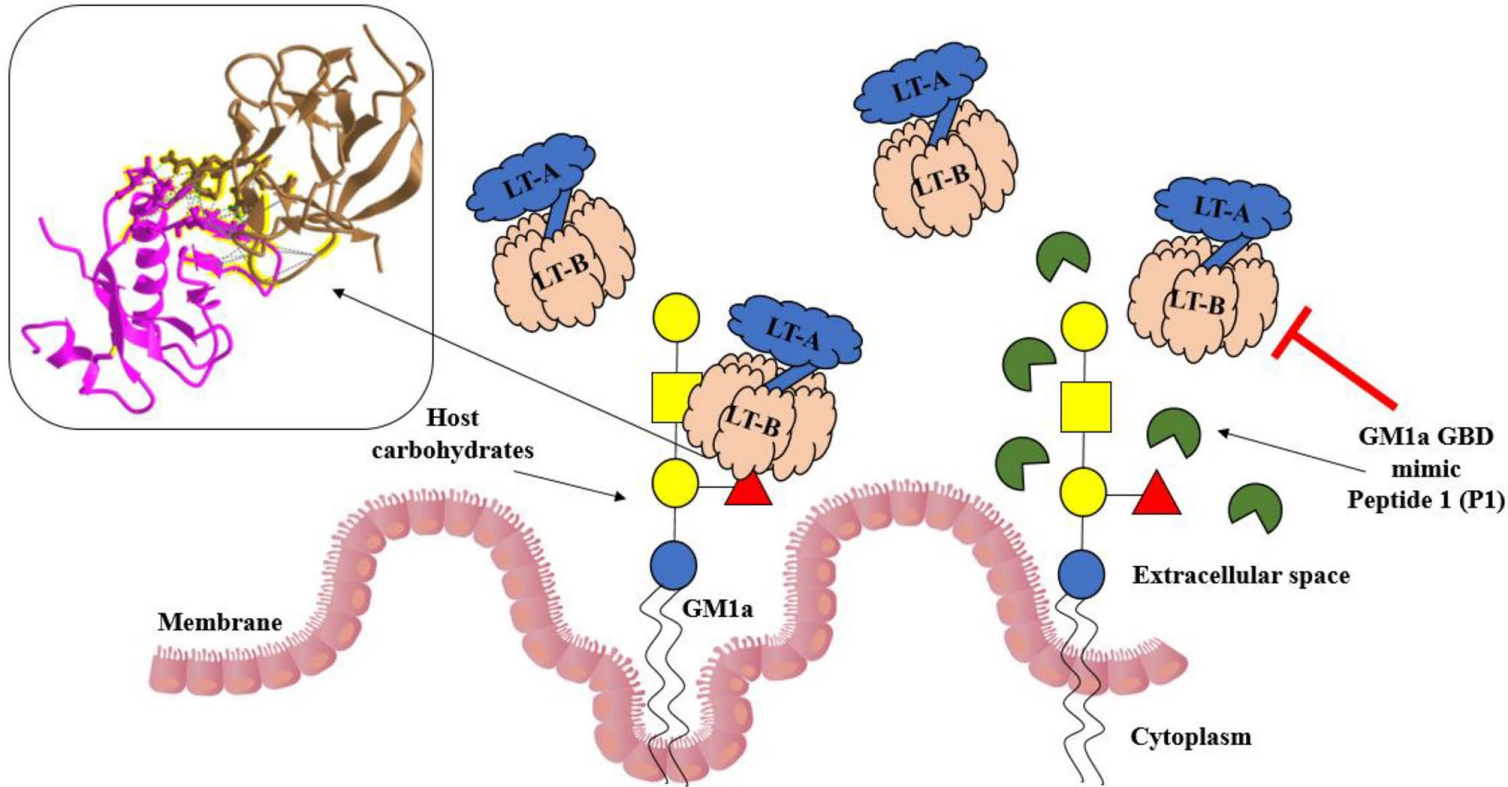
Diagrammatic illustration of an inhibitor-restraining connection in LT-B. Immunotherapy for LT-B might be used to prevent and cure infections by determining the involvement of the GM1a ganglioside-binding domain peptide in the immune response against the pathogen.

In this research, GM1a-GBD replica peptides were developed to inhibit the attachment of LT to GM1a on the surface of the cell membrane. Numerous virulence factors, known as AB5 toxins, are secreted by pathogenic bacteria^46^. The representative LT toxin among them is a hetero-oligomeric holotoxin that belongs to the family of AB5 enterotoxins and is made up of one A subunit and five identical B subunits^47^. Following B subunit attachment to certain host cell surface glycans, cytotoxic A subunits are released into the cytoplasm^48^. Therefore, the GBD of the LT-B subunit plays an important role in cytotoxicity and was the target of our study. We showed that GM1a-like peptides can GM1a GBD by preventing LT-B binding to GM1a. By performing ganglioside microarray and docking simulations, we identified three peptides that effectively prevent LT-B from attaching to GM1a (Figs. 1 and 2). The experimental method used in these results is the ganglioside microarray method. It is mainly used as a microscope glass slide-based glycan microarray and is known to be a very successful and valuable tool for studying lectin–glycan interactions^49^. Moreover, molecular docking simulations are one of the most important methods in the field of peptide modeling^50^. Molecular docking has been recognized as an excellent tool in the study of lectin–glycan ligand complexes to explore specific domains by elucidating interactions and accurately predicting the poses of multiple ligands. Based on these methods, we tried to accurately find the GBD. P1, P2, and P3 are the peptides that were produced by utilizing this approach, with P1 having the most potent inhibitory action on LT-B’s ability to bind to GM1a (Figs. 3 and 4). The reasons why we chose to produce major GBD regions as peptides in this study are as follows. GM1a GBD-peptidomimetics can simulate carbohydrate structures and are easy to produce with today’s currently available peptide synthesis techniques, making them useful antagonists to “block” the cytotoxic effects of LT^51^. A series of studies were conducted to determine whether a synthesized peptide was effective in vitro. LT-B cytokine production by LT-B-induced HCT-8 cells is inhibited by GM1a GBD-mimetic peptide P1, which also inhibits dendritic elongation of intestinal epithelial cells (Figs. 5 and 6). The peptides show great promise as a useful tool for competing with LT-B for GM1a, which is a receptor used in the treatment of cholera and other bacterial diseases (Fig. 7). Because peptides have inherent weaknesses, including physical instability and short circulating plasma half-lives, supplementation is required for their use as therapeutics^52^. It is planned to complement this technology with a focus on peptide drug conjugates and alternative routes of administration^53^. Furthermore, in the intestinal environment, we will conduct in-depth research on delivery processes so peptides can be delivered accurately and effectively^54^. Additionally, in vivo studies using mice have been planned to verify whether peptidomimetics may be administered in an actual intestinal environment. The inhibitors discovered through this research may be developed as novel drugs and may provide data necessary for the development of functional foods that can be actually consumed by humans as well as alternative antibiotic treatments.

In conclusion, we examined how pathogen lectins interact with host gangliosides and glycans early in HCT-8 cell infection. Lectin LT-B was confirmed to bind to the intestinal host ganglioside. Additionally, the effects of synthesizing GM1a GBD-like peptides to inhibit LT-B was tested. Through lectin–glycan interaction studies, we recommend further studies on lectins and glycans to lay the groundwork for developing therapeutic methods for gastrointestinal infections. Our mimic peptide will be promising candidate for preventing toxin-caused diarrhea which has no effective prevention thereby this has great significance in terms of whole world health care.

### Supplementary Information

Supplementary Information 1.

Supplementary Information 2.

## Data Availability

All data that support the findings of this study are available from the corresponding author on reasonable request.
