# Supplementary material for: GM1a ganglioside-binding domain peptide inhibits host adhesion and inflammatory response of enterotoxigenic Escherichia coli heat-labile enterotoxin-B in HCT-8 cells
Source: Sci Rep. 2023 Oct 6;13:16835. doi: 10.1038/s41598-023-44220-5 (PMC10558473; doi:10.1038/s41598-023-44220-5)
Supplement: Supplementary file 1 — Supplementary Information 1. [file 41598_2023_44220_MOESM1_ESM.pdf]

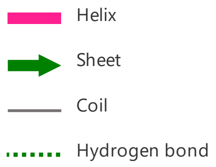

7 hydrogen bond pairs

Atom 1	Atom 2	Distance(Å)
LYS (K) B:23@NZ	ASN (N) B:107@OD1	2.6
LEU (L) B:25@O	GLY (G) B:108@N	3.8
GLU (E) B:29@O	ARG (R) B:26@NH1	2.8
GLU (E) B:29@O	ARG (R) B:26@NH2	2.9
TYR (Y) B:76@OH	ASP (D) B:110@OD1	2.8
GLU (E) B:79@OE1	ASN (N) B:107@ND2	3.2
GLU (E) B:79@OE2	ASN (N) B:107@ND2	2.9

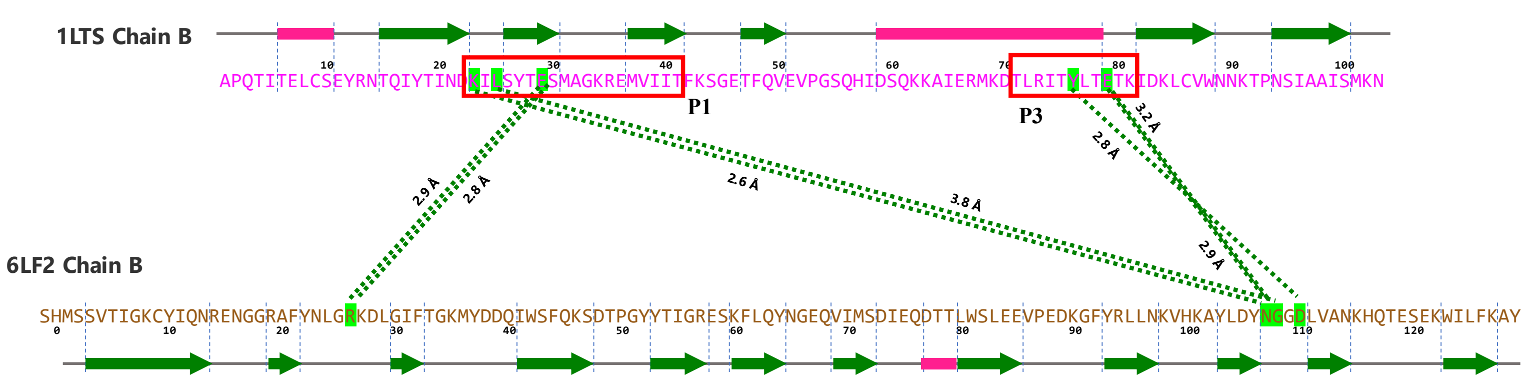

Supplemental data 1A. Performed on all ligands of LT-B and analyzed for hydrogen bonding.

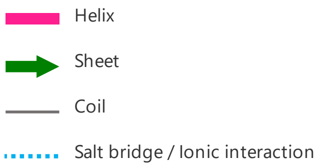

1 Salt bridge / Ionic interaction pairs

Atom 1	Atom 2	Distance(Å)
GLU (E) B:29@OE1	ARG (D) B:26@NH1	4.1

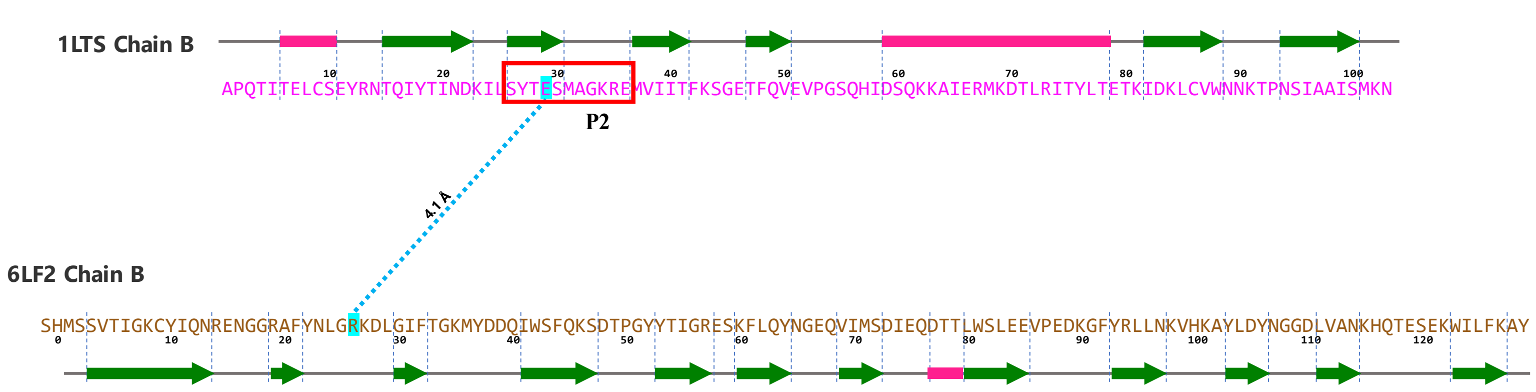

Supplemental data 1B. Performed on all ligands of LT-B and analyzed for ionic bonding.

34 Residue pairs in the contacts

Residue 1	Residue 2	Num Contacts	Min Distance (Å)	C-alpha Distance (Å)
LYS (K) B:23@HZ1	ASN (N) B:107@OD1	5	1.7	10.3
LYS (K) B:23@HZ2	GLY (H) B:108@N	1	3.9	9.4
LEU (L) B:25@CD2	GLU (E) B:15@O	1	3.9	9.4
LEU (L) B:25@O	GLY (G) B:108@CA	3	3.0	5.0
LEU (L) B:25@O	ASN (N) B:107@C	1	3.9	6.6
SER (S) B:26@CA	GLY (G) B:108@C	1	3.6	4.2
SER (S) B:26@CB	ASN (N) B:16@ND2	1	3.4	7.0
SER (S) B:26@CB	ILE (I) B:31@CG2	1	3.7	6.9
SER (S) B:26@OG	THR (T) B:33@HG1	1	3.7	7.6
TYR (Y) B:27@N	GLY (G) B:109@N	1	3.9	4.6
TYR (Y) B:27@H	GLY (G) B:108@C	1	3.4	6.0

Residue 1	Residue 2	Num Contacts	Min Distance (Å)	C-alpha Distance (Å)
TYR (Y) B:27@O	ILE (I) B:31@H	1	3.4	6.9
THR (T) B:28@H	THR (T) B:33@HG1	1	4.0	6.3
THR (T) B:28@CA	ILE (I) B:31@O	1	4.0	5.9
THR (T) B:28@CB	GLY (G) B:34@H	1	4.0	7.5
THR (T) B:28@OG1	PHE (F) B:32@CA	1	3.5	5.1
THR (T) B:28@CG2	PHE (F) B:21@CZ	1	4.0	9.1
GLU (E) B:29@OE2	ARG (R) B:26@HH22	4	1.9	9.7
GLU (E) B:29@H	PHE (F) B:21@CZ	1	3.3	10.2
SER (S) B:30@N	ARG (R) B:26@HH21	1	3.2	9.4
SER (S) B:30@CB	PHE (F) B:32@CZ	1	4.0	8.7
SER (S) B:30@CB	TYR (Y) B:37@OH	1	3.6	10.3

Residue 1	Residue 2	Num Contacts	Min Distance (Å)	C-alpha Distance (Å)
MET (M) B:31@SD	ARG (R) B:26@H	1	4.0	8.9
ALA (A) B:32@H	TYR (Y) B:37@HH	1	3.6	9.2
ARG (R) B:35@CB	TYR (Y) B:37@OH	1	3.1	10.7
ARG (R) B:35@CG	LYS (K) B:35@CD	1	3.8	8.3
ILE (I) B:39@CB	THR (T) B:33@OG1	1	3.6	6.5
ILE (I) B:39@CB	GLY (G) B:34@H	1	3.3	6.1
ILE (I) B:39@CD1	PHE (F) B:32@CE1	1	3.8	7.9
THR (T) B:41@OG1	THR (T) B:33@CB	1	4.0	7.4
TYR (Y) B:76@CD1	GLY (G) B:108@CA	1	4.0	6.8
TYR (Y) B:76@CD1	GLY (G) B:109@H	1	3.6	8.4
TYR (Y) B:76@HH	ASP (D) B:110@OD1	5	1.9	10.3
GLU (E) B:79@OG1	ASN (N) B:107@HD22	4	2.0	9.0

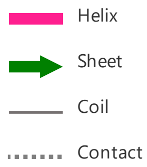

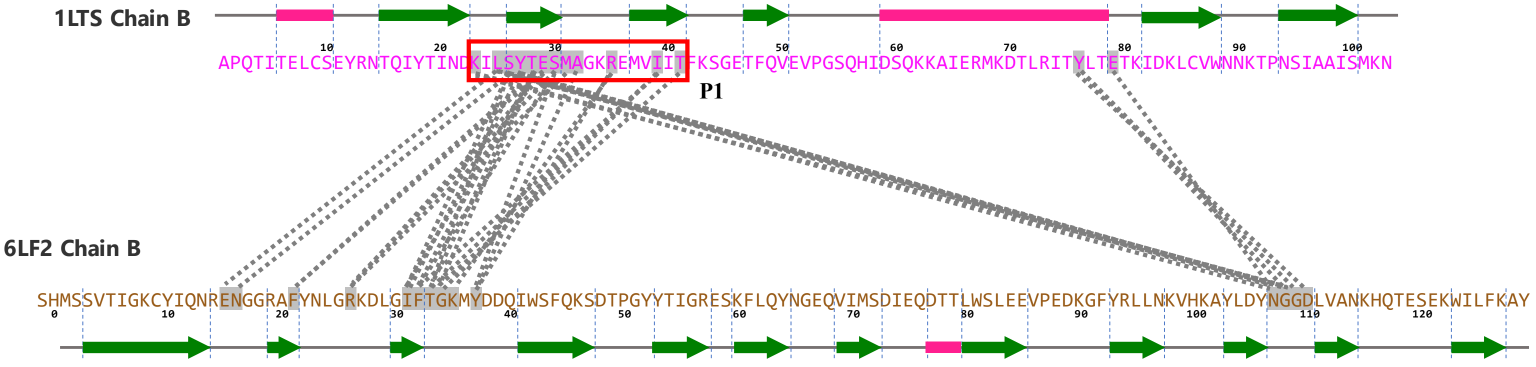

Supplemental data 1C. Performed on all ligands of LT-B and analyzed for general bonding strength.

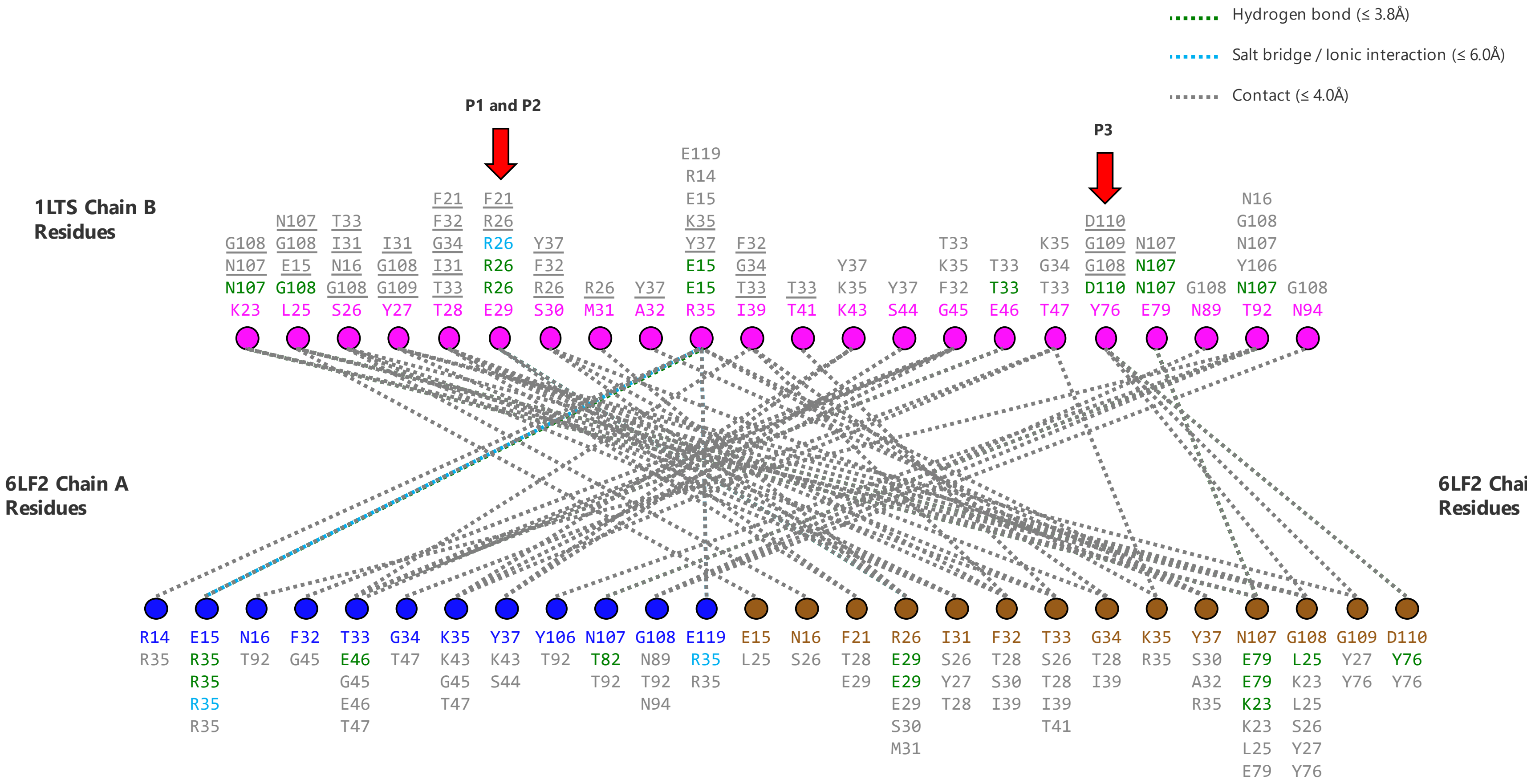

Supplemental data 1D. Predicted binding sites for three GM1a GBDs (KILSYTESMAGKREMVIIT, SYTESMAGKRE and TLRITYLTETK)
