# Supplementary material for: GM1a ganglioside-binding domain peptide inhibits host adhesion and inflammatory response of enterotoxigenic Escherichia coli heat-labile enterotoxin-B in HCT-8 cells
Source: Sci Rep. 2023 Oct 6;13:16835. doi: 10.1038/s41598-023-44220-5 (PMC10558473; doi:10.1038/s41598-023-44220-5)
Supplement: Supplementary file 2 — Supplementary Information 2. [file 41598_2023_44220_MOESM2_ESM.pdf]

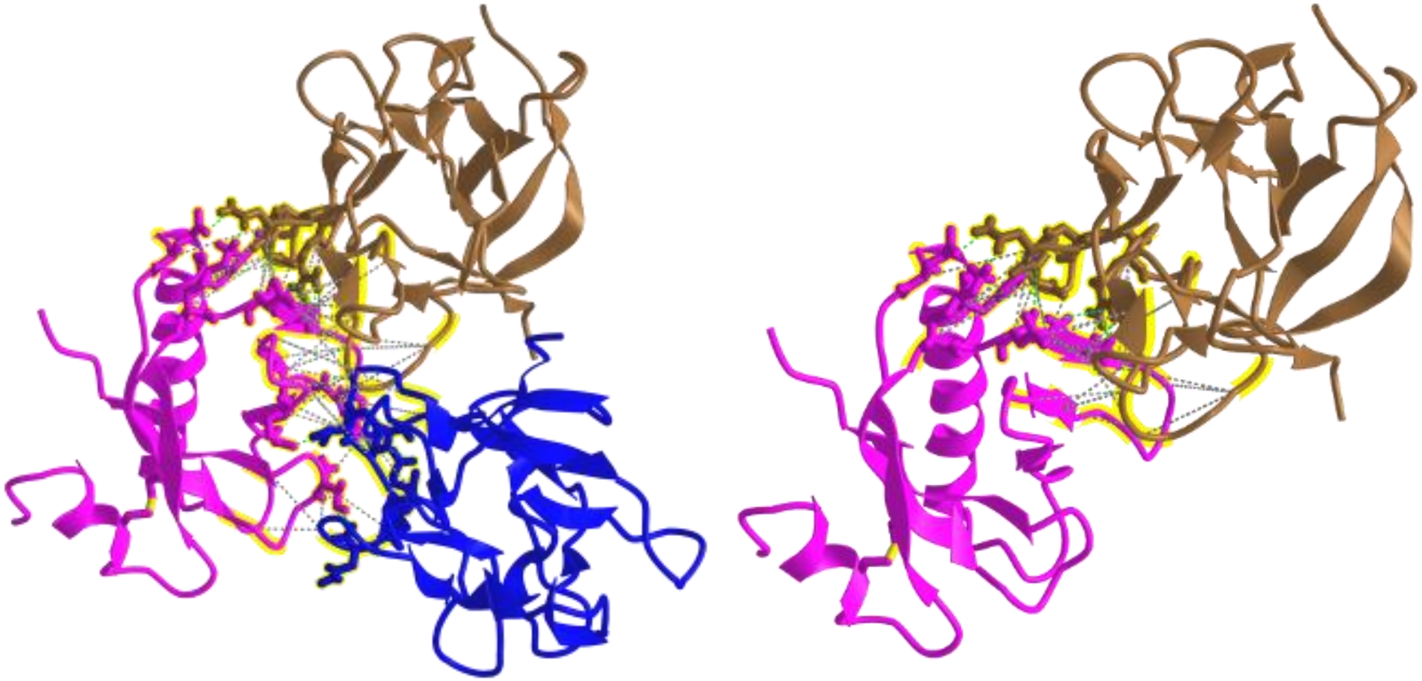

	hybrogen bond pairs
	salt bridge / ionic interaction pairs
	Residue pairs in the contacts

11	hybrogen bond pairs
3	salt bridge / ionic interaction pairs
53	Residue pairs in the contacts

7	hybrogen bond pairs
1	salt bridge / ionic interaction pairs
34	Residue pairs in the contacts

Supplemental data 2. The docking binding form of GM1a and LT-B.
